# Catalyzing sustainable development: insights from the international workshop on STI policies and innovation systems in Central America

**DOI:** 10.3389/frma.2024.1511393

**Published:** 2024-12-11

**Authors:** Jorge A. Huete-Pérez, Alma Cristal Hernández-Mondragón, Douglas S. Massey, Luz M. Cumba García, Bernard Amadei, Nadia De León Sautú, Maria L. Acosta, Omar Asensio, John Boright, Serena Cosgrove, Emilio Hernández Hernández, María López-Selva, Juan L. Manfredi, Fanor Mondragón, José M. Natera, Oscar C. Picardo Joao, Angelo Rivero Santos, Harold O. Rocha

**Affiliations:** ^1^Science, Technology and International Affairs Program, Georgetown University, Washington, DC, United States; ^2^Department of Research and Multidisciplinary Studies, Center for Research and Advanced Studies, National Polytechnic Institute of Mexico (CINVESTAV), México City, Mexico; ^3^Sociology and Public Affairs, Princeton University, Princeton, NJ, United States; ^4^Science and Technology Policy Fellowships, American Association for the Advancement of Science, Washington, DC, United States; ^5^Department of Civil, Environmental and Architectural Engineering, College of Engineering and Applied Science, University of Colorado Boulder, Boulder, CO, United States; ^6^Educational Research Center of Panama, Panama, Panama; ^7^Department of Law, University of Central America, Managua, Nicaragua; ^8^Academy of Sciences of Nicaragua, Managua, Nicaragua; ^9^Institute for the Study of Business in Global Society, Harvard Business School, Boston, MA, United States; ^10^School of Public Policy, Georgia Institute of Technology, Atlanta, GA, United States; ^11^International Affairs, National Academy of Sciences, Washington, DC, United States; ^12^International Studies, Seattle University, Seattle, WA, United States; ^13^Consultative Group to Assist the Poor, Washington, DC, United States; ^14^Research Institute in Natural Sciences and Technology, Universidad Rafael Landívar, Guatemala, Guatemala; ^15^Walsh School of Foreign Service, Georgetown University, Washington, DC, United States; ^16^Institute of Chemistry, University of Antioquia, Medellín, Antioquia, Colombia; ^17^Institute of Economic Research, National Autonomous University of Mexico, México City, Mexico; ^18^Institute of Science, Technology and Innovation, Francisco Gavidia University, San Salvador, El Salvador; ^19^Center for Latin American Studies, Walsh School of Foreign Service, Georgetown University, Washington, DC, United States; ^20^Law School, University of Wisconsin-Madison, Madison, WI, United States

**Keywords:** STI policy, science-diplomacy, Central America, sustainable development, capacity building

## Abstract

This article examines the landscape of Science, Technology, and Innovation policies in Central America, focusing on Nicaragua, Guatemala, Honduras, and El Salvador. These nations face significant challenges in leveraging STI for sustainable development, including financial constraints and limited resources. Additionally, Central America struggles with systemic issues such as corruption, violence, and high levels of emigration, further complicating efforts to advance STI. A workshop organized by Georgetown University's Science Technology and International Affairs program brought together scholars to discuss STI policies, resulting in key recommendations. The article highlights critical challenges, including over-reliance on state funding, stagnant researcher numbers, and the pressing need for research diversification. It emphasizes the importance of youth engagement, leadership, and resilience in shaping effective STI policies. Recommendations include investing in science education, establishing governmental scientific advisory bodies, promoting research diversity, and addressing climate change through STI strategies. The findings provide valuable insights for scholars, policymakers, and international organizations working with less developed nations globally.

## Introduction

In the pursuit of fostering social and economic progress, especially in developing nations, the role of scientific and technological advancements is pivotal. Some Latin American countries, over the past few decades, have proactively devised and implemented public policies to encourage the adoption and dissemination of scientific innovations, thereby stimulating domestic technological transformations (Crespi and Dutrénit, 2014). However, the less developed countries in Central America such as Nicaragua, Guatemala, Honduras, and El Salvador, grapple with an array of challenges, encompassing financial constraints, inadequate infrastructure, a scarcity of human resources, and the absence of crucial institutions and policies (Obinna, 2019; Appolloni, 2009).

In this context, Central American countries face difficulties in effectively harnessing expertise and achievements in Science, Technology, and Innovation (STI) due to limited resources and inefficient national innovation systems, which emerges as a primary hurdle (Casalet and Buenrostro, 2014). Despite recent initiatives and dialogues with international organizations, these smaller nations have received limited academic attention, with most studies concentrating on the larger, more industrialized countries in the region.

To tackle these challenges and with the ultimate goal to pave the way for accelerated sustainable growth and improved living standards in the Central American region, the Science Technology, and International Affairs program of Georgetown University (STIA), took the initiative to organize an international workshop. The workshop served as a platform for scholars from Central America, Colombia, Venezuela, Mexico, Spain, and the US to deliberate on the current state of STI policies and scientific development in the region. By focusing on five general topics, including the status of STI policies and scientific development, the evolving role of STI policies in Latin America, empowering the next generation of scientists, building resilient innovation ecosystems for sustainable development, and perspectives from international scientific societies and funding institutions, the workshop aimed to delve into the intricacies of STI policies and scientific progress in Central America.

The findings presented in this article stem from the discussions held during the workshop. These insights aim to inform policymakers, scholars, and practitioners on the development of more robust and effective STI policies in Central America, contributing to the broader discourse on science governance and diplomacy. Furthermore, the recommendations generated offer valuable guidance for international organizations and development agencies working with less developed countries, highlighting the global relevance of the challenges and potential solutions discussed.

The following section introduces the key topics discussed during the workshop, covering the history of science and technology policies in the region, their current state, and the future perspectives identified.

## The challenge of doing science in Latin America

### Colonial foundations and post-independence legacy

Latin America was created by the colonization of large portions of the Americas by Spain and Portugal in the early 16th century, yielding one Portuguese-speaking nation (Brazil) and 16 Spanish-speaking nations scattered across Central America, South America, and the Caribbean (Crowley and Roger, 2016; Fernández-Armesto and Giraldo, 2024). The Portuguese and Spanish Empires were constructed as extractive systems designed to transfer wealth and resources from the colonies to the crown, laying the institutional foundations for the later evolution of economies based on the export of commodities rather than the autonomous production of goods and knowledge. Power was concentrated in a centralized viceregal bureaucracy whose power was guaranteed by imperial troops to ensure the continued flow of wealth to the royal houses of Europe.

The systems of extraction that emerged from this common beginning varied from place to place depending on geography, climate, the character of the indigenous population, and over time on the degree of European settlement and participation in the Atlantic Slave Trade. While these colonial powers often focused on extraction and exploitation, they also established universities and other institutions of higher learning, though access to these institutions was primarily limited to the elite. Nonetheless, in the years since achieving independence in early 19th century, it has proved difficult for Latin American nations to escape the political legacy of autocracy and the economic legacy of commodity export to produce democratic regimes capable of independently producing goods, services, and knowledge salable in the global economies that emerged first in the late 19th and early 20th centuries, and more recently in the late 20th and early 21st centuries (Weaver, 2018).

### Current challenges

A survey of indicators of economic development, internal investment, freedom, the rule of law, exposure to violence, and state fragility reveals a diversity of outcomes across the region (Massey, 2023). Over the years, some countries, such as Argentina, Chile, Uruguay, and Costa Rica, have made significant progress toward democratic governance and economic development, though they continue to grapple with the legacies of colonialism, authoritarianism, and inequality. Others remain mired in authoritarian governance and economic dependence on the export of a limited range of commodities (e.g., Cuba, Honduras, Nicaragua, and Venezuela).

Whatever their relative success within the region, compared to nations in Europe and North America, to date most Latin American countries have not been very successful in creating the institutional structure to enable meaningful contributions to the global, information-based knowledge economy of the 21st century, which requires significant public and private investment in science and technology (Petras and Veltmeyer, 2014).

### Pathways to progress

Given the historical heterogeneity of the national trajectories that emerged from common origins in Spanish and Portuguese colonialism, there can be no simple formula for development applicable uniformly throughout the region. Progress must begin with a thorough understanding of each nation's social, political, and economic history, and the configuration of formal institutions and informal practices that have resulted from that history. Moreover, addressing the issue of corruption, which has plagued many countries in the region, is essential for sustainable development (Mauro, 1995; Seligson, 2002). Building on such an understanding, policymakers can then work to develop the political strategies and organizational practices required to adapt existing social and economic structures to better foment scientific research and sustain the technological innovation needed for growth in the post-industrial economy of the 21st century.

## The resilience of Central American ethnic communities

Indigenous communities in Latin America struggle disproportionately with poverty and social exclusion (Davis, 2002). Central American ethnic communities confront unique challenges amidst the region's socio-political complexities, particularly the prevalence of violence in Honduras, El Salvador, Guatemala, and Nicaragua (Jesus and Hernandes, 2019). The Nicaraguan government's plans to build an inter-oceanic canal raise concerns about the impact on indigenous rights (Berryhill, 2015) and the environment (Huete-Pérez et al., 2016). To ensure that the benefits of development projects are equitably distributed and that they reach marginalized communities, it is crucial to adopt inclusive and participatory development processes that prioritize the voices and needs of local communities. In this context, assessing the readiness for rebuilding innovation systems becomes crucial. Indigenous environmental defenders, who often operate under challenging and underreported risks, play a significant role in these efforts. Moreover, funding dynamics must align with the goal of establishing a stable societal foundation, one that supports scientific and technological advancement while safeguarding these communities' contributions to sustainable development.

Social, environmental, and sustainability research plays a key role, revealing ecological and cultural impacts. Governments must address challenges faced by indigenous communities, necessitating nuanced governance approaches. Policies should extend beyond innovation systems, fostering an inclusive, sustainable, and culturally sensitive environment. Governance structures must engage with underreported risks, ensuring enforcement of international human rights laws. Effective policies should address environmental changes, support modern tools for communication, and tackle critical issues such as undisclosed concessions within indigenous territories, promoting transparency and accountability. This holistic approach aims to empower Central American ethnic communities in the face of multifaceted challenges.

## Navigating the challenges and opportunities of science, technology, and innovation

Central America aspires to build a robust STI ecosystem to drive sustainable development and improve the lives of its citizens. However, the region faces intricate challenges that demand nuanced policy responses and strategic interventions.

## A common thread of obstacles

Central America's Science, Technology, and Innovation (STI) landscape faces intricate challenges demanding nuanced policy responses. Current limitations, including heavy reliance on state funding for universities, hinder the implementation of effective institutional models (Padilla-Pérez and Gaudin, 2014). The prevalence of Medical Sciences underscores the necessity for diversification in research pursuits, especially given stagnant researcher numbers.

Central America is grappling with intricate challenges in the realm of Science, Technology, and Innovation (STI). From institutional dismantling in Nicaragua (Puig and Serra, 2020) to educational obstacles in El Salvador and environmental issues in Guatemala, the region faces multifaceted hurdles that demand urgent attention. These challenges encompass issues such as limited local prospects in Panama, structural educational problems hindering scientific development in El Salvador, and the need for strategic focus on high-level education in Guatemala.

Roles within National Innovation Systems spotlight the state's funding responsibility, businesses supporting industry-related research, and universities contributing to societal benefit. However, aligning STI policies with broader development goals necessitates strategic reevaluation. Central America's resource constraints call for tailored STI resources, urging the formulation of robust policy frameworks.

The analysis illuminates multifaceted challenges, stressing the critical role of financial investment, the need for tailored policy frameworks, and the imperative of addressing systemic issues for innovation and sustainable development in Central America.

## Country-specific issues

While Central America faces common regional challenges in Science, Technology, and Innovation (STI), each country grapples with unique obstacles and opportunities that shape its individual STI landscape.

### Guatemala

Guatemala faces pressing challenges in STI, exacerbated by an economic model harming natural elements vital for health and agriculture, particularly deforestation (with 12% of tree cover loss from 2001 to 2023 driven by deforestation itself, according to data from the Landsat Program, processed by researchers at the University of Maryland) and water access (with most rural areas lacking improved sources and only 70% of the population having basic drinking water). A study by Bullock et al. (2020) found that deforestation and forest degradation are significant issues in Guatemala's protected areas. The study indicates that the combined factors (broader impact of forest disturbance, including degradation and natural disturbances) have significantly impacted the protected areas, with some experiencing disturbance rates exceeding 95%. This underscores the urgent need for environmentally focused STI initiatives to combat deforestation and improve water management. To address these challenges, Guatemala needs a multi-faceted approach that combines technological innovation with social and economic reforms. While a skilled workforce is essential for driving sustainable development, it is equally important to address underlying issues such as corruption, inequality, and weak governance. Creating opportunities, jobs, and investing in all levels of education, particularly in science and engineering, can help equip the country with the necessary human capital to develop and implement innovative solutions.

### Honduras

Honduras faces significant hurdles in its scientific and technological advancement (Bonilla et al., 2022). Decades of insufficient funding and inconsistent government policies have hampered progress. The National System of Science, Technology, and Innovation (SNCTI) lacks integration between government entities, universities, and other sectors, further hindering effective development. While initiatives like Honduras Global (HG) engage the scientific diaspora, broader policies are needed to fully leverage their expertise.

### El Salvador

El Salvador has seen a shift toward procedural democracy in the last few decades, with competitive political parties and peaceful transfers of power. However, further efforts are crucial to strengthen institutional protections for indigenous citizens (Gellman and Bellino, 2019). While this political progress is noteworthy, the country faces significant challenges in its educational and scientific development. Structural educational issues, such as low quality and inadequate teaching standards, obstruct scientific development in El Salvador (Picardo Joao et al., 2020; Picardo Joao, 2004). A preference for migration over higher education due to better remuneration prospects creates socio-economic ramifications.

### Nicaragua

The institutionalization of science in Nicaragua began in the 1980s with strong support from Nordic countries, particularly Sweden. The 1990s saw further development with the creation of research institutes within public and private universities, particularly the University of Central America (UCA). However, as a developing nation facing poverty, research often focused on immediate challenges, hindering long-term strategic initiatives. Now, the situation is dire. A sociopolitical crisis fueled by reported government abuses [(United Nations High Commissioner for Human Rights (UNCHR), 2024)] has devastated Nicaragua's academic landscape. The closure of over half its universities, including UCA and the Academy of Sciences, has crippled scientific infrastructure and threatens academic freedom (Karath, 2023). Restoring academic freedom, fostering international collaboration, and strategically investing in research focused on long-term sustainability are crucial steps for Nicaragua to rebuild its scientific capabilities and address its pressing challenges.

### Costa Rica

Despite its robust scientific infrastructure and notable integration of STI policies into national development plans, Costa Rica faces challenges in fully capitalizing on science and technology for social and economic progress. The country grapples with substantial infrastructure deficiencies, particularly in transportation and water treatment, further strained by a significant fiscal deficit. Being a small, open economy, Costa Rica is highly vulnerable to external shocks like global inflation, weakened global growth, and tightened financing. Climate vulnerabilities, intensified by El Niño, add to these uncertainties. However, Costa Rica stands out from its Central American peers. It has a thriving startup scene across diverse sectors like software development, artificial intelligence, renewable energy, and biotechnology (Jarquin-Solis and Mauduit, 2021).

### Panamá

Panamá's recent science diplomacy success offers a model for Central America. By integrating scientific advice, they tackle complex regional challenges in health, agriculture, and the environment. This leadership paves the way for other small countries to excel in science diplomacy. However, Panamá grapples with a trend where PhD graduates seek opportunities abroad due to limited local prospects. Challenges include external funding dependence, limited research engagement in universities, and a critical lack of funding from the business sector. Weak governance calls for collaborative efforts to fortify the STI system (Gittens et al., 2021).

## National innovation systems and regional dynamics of STI in Central America

Following the challenges faced by each Central American country, as outlined above, it is important to explore the broader frameworks and emerging trends shaping STI in the region. Countries such as Costa Rica and Panama offer more established systems that could model pathways for others, yet Central America as a whole requires deeper regional collaboration and policy consistency to overcome shared barriers to STI-driven sustainable development.

### National innovation systems

Central American countries present contrasting scenarios in their STI development. While Costa Rica and Panama, have established relatively robust STI frameworks, others struggle to sustain systems that can effectively support scientific research and innovation. Institutional structures for science and technology were set up across Central America starting in the early 1990s, with Costa Rica (1990), Guatemala (1991), El Salvador, and Panama (1992), and Honduras (1993) enacting regulations to create foundational innovation systems (Viales-Hurtado et al., 2021). Nicaragua, however, was slower to act, with a research promotion structure established in 1995, only becoming operational in 2000 (Huete-Pérez, 2008). By contrast, most other Latin American countries had already developed National Research Councils by the 1950s (Crespi and Dutrénit, 2014).

Today, Costa Rica leads the region with a mature National Innovation System supported by policies that foster innovation across public and private sectors, particularly in clean energy and biodiversity conservation research. Similarly, Panama has established a robust framework through its National Secretariat of Science, Technology, and Innovation (SENACYT), overseeing policies that emphasize knowledge creation in health and agriculture and benefiting from both government funding and foreign investment. In contrast, Nicaragua, Guatemala, Honduras, and El Salvador share common structural challenges, including institutional fragmentation, limited research and development (R&D) funding, and insufficient policy continuity (Padilla Pérez et al., 2012). Their STI activities remain concentrated in under-resourced government agencies and a few public universities, relying heavily on regional initiatives to supplement national capacity (Bovenschulte, 2010).

### Intersectoral collaboration

The effectiveness of intersectoral collaboration varies significantly across the region, with Costa Rica and Panama demonstrating relatively strong cross-sector partnerships. Costa Rica's private sector actively contributes to STI funding, particularly in clean energy and biotechnology, while Panama has formed alliances with international research centers that align academic research with industry needs. This cross-sectoral engagement bolsters the scientific capacity of these countries and provides a model for leveraging both domestic and foreign expertise.

In contrast, intersectoral collaboration in Nicaragua, Honduras, El Salvador, and Guatemala is more limited, with government and academic institutions often working in isolation from the private sector (Viales-Hurtado et al., 2021). This limited coordination leads to mismatched priorities between STI initiatives and community or industry needs. However, regional organizations such as the Central American Integration System (SICA) and the Economic Commission for Latin America and the Caribbean (ECLAC) have introduced collaborative frameworks to strengthen partnerships and resource-sharing across the region (Peralta Quesada and Padilla Pérez, 2019). These frameworks aim to build unified research agendas that reflect the region's needs and are essential for tackling shared challenges in areas such as climate change and public health.

### Policy and institutional frameworks

Policy frameworks across Central America reflect diverse levels of engagement and resource allocation toward STI. Costa Rica and Panama have adopted well-coordinated policies, including tax incentives for research and development, which attract investments and drive innovation. In contrast, other countries continue to face policy inconsistencies and limited budgets. Addressing these issues, SICA and ECLAC advocate for consistent STI policy integration across Central America, with the Central American Innovation Agenda pushing for policy support in areas of strategic importance such as climate adaptation, STEM education, and health (Padilla, 2013).

Science diplomacy has also emerged as a key strategy, particularly for resource-constrained nations. Through foreign partnerships, Honduras and Nicaragua may gain access to technical expertise and advanced research that supports national STI goals, helping address critical gaps in health and environmental resilience.

### Emerging trends

Across Central America, emerging trends indicate promising advancements in STI. Digital transformation and increased investment in biotechnology and renewable energy are gaining momentum, especially in Costa Rica, Nicaragua and Panama. The region is also seeing a growing emphasis on research diversity and climate adaptation strategies, with initiatives that recognize the value of indigenous knowledge systems as part of sustainable development.

As these trends take shape, regional integration remains essential to ensure equitable progress across countries with different levels of STI maturity. The initiatives in Costa Rica and Panama illustrate effective models that other nations can draw from, while regional organizations provide the frameworks needed to drive collective action and overcome persistent challenges like funding constraints, institutional fragmentation, and policy inconsistencies. This integrated approach is crucial for building a resilient, STI-driven future across Central America.

## Fostering opportunities for early career researchers in Central America

Recognizing the critical role of human capital, the workshop also explored ways to empower early career researchers (ECRs) in Central America. A strong scientific workforce is fundamental for harnessing Science, Technology, and Innovation (STI) for sustainable development. This section delves into the GloSYS project by the Global Young Academy (Nieto et al., 2022).

GloSYS, extending beyond a global initiative, carries specific relevance for Central America's scientific landscape. It thoroughly investigates the educational, experiential, and aspirational dimensions influencing ECRs in the region, identifying critical constraints such as job precarity, financial insecurity, bureaucratic hurdles, language prominence, and the intricate balance between career and personal goals. Furthermore, it is essential to address these challenges by increasing access to higher education, providing adequate funding for research, and creating supportive research environments that encourage innovation and critical thinking, factors that are relevant to foster a thriving research culture in the region.

ECRs are caught in a double bind: a brutal job market and crippling financial insecurity. Short-term contracts, fierce competition, and stagnant salaries leave them stressed, uncertain, and often questioning their abilities. Their passion for research clashes with the harsh reality of a system that fails to support their scientific ambitions. This situation demands immediate attention and policy changes to nurture the next generation of scientific leaders in the region and beyond (Nieto et al., 2022). To cultivate a prosperous scientific culture, it is essential to address systemic challenges such as inadequate infrastructure and a lack of mentorship opportunities. Nurturing interdisciplinary collaboration and promoting public engagement are essential steps toward creating an environment that fosters innovation, critical thinking, and scientific excellence.

The GYA's leadership program for early career scientists, exemplified by an event in Leticia, Colombia in December 2022, stands out as notably significant. Held strategically outside a capital city, this highlights the project's commitment to diverse and inclusive representation, crucial in regions like Central America (Rondón-Jara et al., 2024).

The most recent GloSYS report meticulously addresses the challenges confronting ECRs in Latin America and the Caribbean. Issues like underfunded research systems and deficient infrastructure contribute to problems such as the absence of permanent employment prospects and non-remunerated work. The report doesn't merely diagnose these challenges; it also proposes mitigation strategies.

GloSYS, an ongoing research initiative, actively seeks collaboration with academic and research institutions in the region, extending an invitation to young scientists in Central America to play an active role in shaping their careers and contributing to the broader global scientific discourse. As the project advances, its outcomes have the potential to inform policies and initiatives that positively impact the trajectories of early career researchers in Central America.

By fostering a more supportive environment for ECRs, as outlined by GloSYS' recommendations, Central American nations can harness the full potential of their scientific workforce for sustainable development.

## A path forward: key actions for a sustainable future based on STI

Based on the findings from the international workshop, we present the following roadmap as a comprehensive overview of the current state of STI in Central America, along with actionable recommendations to address key challenges and opportunities. These insights aim to guide policymakers and stakeholders in developing robust strategies to overcome the region's specific barriers to STI progress. The main findings have been categorized into four key areas—structural challenges, sociopolitical and environmental issues, education and human capital development, and innovation and energy transformation—and are outlined in Tables 1–4. Notably, a discussion on the critical issue of budget allocation in universities across the region is included, highlighting that the majority of funds are directed toward teaching rather than research. This imbalance represents a significant structural challenge to advancing STI capabilities in these nations. Each table provides targeted recommendations that address the unique needs and opportunities within these categories, offering a clear path forward for sustainable development in the region.

**Table 1:** Structural challenges and innovation systems in Central America.

**Challenges**	**Actionable interventions**
Lack of institutional framework for global knowledge economy participation	STI policies should account for historical and social contexts, adapting structures to promote research and sustain innovation for post-industrial growth
Insufficient financing, poor institutional coordination, limited academia-industry collaboration, and inadequate STI indicators	Prioritize investments in STI with adequate state funding and coordinated fiscal policies. Engage governments, trade groups, and civil society to align efforts with the UN SDGs through impact investments by 2030. Explore sectors beyond Medical Sciences to include biotechnology
Limited understanding of innovation systems in less developed countries, including the transition from traditional goals toward broader national challenges	Realign STI policies to target complex national issues, adopting frameworks tailored to regional realities. Develop comprehensive policies to drive innovation, strengthening institutional frameworks and stakeholder coordination
Insufficient public investment in science and research, hindering the development of knowledge societies in Central America	Central America should allocate a minimum of 1% of GDP for science and technology. Each country should establish at least one research university and create national research institutes regionally. Collaborative science and technology parks with universities are vital for strengthening innovation ecosystems
Disproportionate allocation of university budgets toward teaching over research in many Central American countries	Implement policies to gradually increase the proportion of university budgets allocated to research activities, while maintaining educational quality. Encourage partnerships with industry and international research institutions to supplement research funding

**Table 2:** Sociopolitical and environmental challenges in Central America.

**Challenges**	**Actionable interventions**
Sociopolitical challenges, such as migration, gender violence, and authoritarianism, which undermine democratic and economic development	Promote collaborative research and science diplomacy, leveraging global partnerships for STI. Focus on evidence-based policymaking to address root causes of migration and violence, particularly in the Northern Triangle
Censorship and attacks on environmental scientists and activists, particularly in regions like Nicaragua's Mayangna Sauni. As territory, which weaken scientific systems and limit investment	Develop national policies to protect environmental scientists, activists, and vulnerable communities. Strengthen law enforcement and prioritize indigenous rights in Mayangna Sauni As. Nicaraguan authorities must uphold Inter-American Human Rights protections, ensuring culturally sensitive consultations with indigenous populations
Transition from a destructive, business-as-usual model to one driven by STI for sustainable development	Establish robust strategies for sustainable growth, focusing on education, climate resilience, and Ph.D. initiatives. Strengthen regional collaboration and prioritize comprehensive planning for environmental and societal challenges

**Table 3:** Educational and human capital development.

**Challenges**	**Actionable interventions**
Disconnection between the scientific community and policymakers, hindering the development of effective solutions	Establish governmental scientific advisory bodies to bridge scientists and policymakers. Provide training at the science-policy interface to improve mutual understanding and communication
Nicaragua faces severe threats to academic freedom and institutional autonomy, with the closure of over half its universities, including the Jesuit University of Central America and the Academy of Sciences of Nicaragua	Implement measures to safeguard academic freedom and institutional autonomy in Nicaragua. Focus on reopening universities, including the Jesuit University of Central America, and ensuring long-term stability in higher education. In doing so, there may be opportunities for science diplomacy to play a role in fostering dialogue and cooperation between relevant stakeholders in the region
Low educational quality, high dropout rates, and insufficient state policies in scientific and technological education	Implement long-term educational reforms to improve school quality and retention rates. Ensure sustained investments in science education across Central America
Lack of early-career scientist engagement in policymaking due to limited awareness of opportunities and resources	Encourage scientists to join fellowships and professional societies that offer policy training. Support community outreach, science communication, and evidence-informed policymaking

**Table 4:** Innovation and energy transformation in Central America.

**Challenges**	**Actionable interventions**
Immense energy challenges as demand is forecasted to grow significantly by 2050	Develop STI policies in areas like smart metering and digital transformations in the energy sector. Promote energy efficiency and conservation in buildings and transportation through sector-specific energy plans and technology standards
Agricultural technology focused on high-income countries, reducing its effectiveness in addressing Central American needs	Redirect agricultural investments to address local challenges and ensure that agricultural technology aligns with the region's specific needs. Funders (both public and private) should play a vital role in supporting this transformation

Furthermore, to foster an environment conducive to STI advancement in Central America, comprehensive policy reforms are urgently needed. These reforms should prioritize investments in education—particularly at the graduate level—and involve curriculum updates that emphasize research skills and critical thinking. Additionally, policies should focus on creating supportive research environments through adequate funding, mentorship, and opportunities for collaboration. Adopting a holistic approach that addresses the multifaceted challenges facing the region is essential, considering the following areas:

### At the core of this strategy is the transformation of the educational landscape

Policymakers must focus on improving teaching standards and quality across all educational levels, with a particular emphasis on incentivizing higher education in Science, Technology, Engineering and Math (STEM) fields. This educational revitalization should be coupled with the creation of research-oriented technological hubs and centers nationwide, bridging the gap in graduate programs focused on scientific research.

### Collaboration is key to this transformation

Fostering partnerships between Central American countries, incentivizing student exchanges, and developing connections with international institutions can significantly enhance knowledge transfer and STI capabilities. Moreover, a comprehensive policy promoting public-private partnerships is essential. This approach will encourage collaboration between universities, research institutions, private sector companies, and international organizations, driving the development and implementation of innovative technologies crucial for addressing pressing issues such as environmental sustainability.

### Government action plays a pivotal role in this roadmap

Prioritizing research through concrete measures and investments in education can propel national progress and prosperity. Additionally, offering fiscal incentives for companies investing in innovation could catalyze private sector involvement in STI development. To implement these recommendations effectively, we propose the following specific actions:

### Prioritizing research

Establishing a National Research Fund that allocates a dedicated percentage (e.g., 1%−2%) of the annual national budget to research initiatives is a key step. This fund should be overseen by an independent council of experts responsible for evaluating and distributing resources based on national priorities and scientific merit. Additionally, implementing a competitive grant system can promote innovative projects aligned with the country's development goals, ensuring that funding is directed toward impactful initiatives.

### Investing in education

To cultivate a robust STI ecosystem, it is imperative to invest in education and research. A comprehensive STEM education strategy, from primary to tertiary levels, is essential to nurture a skilled workforce. This includes introducing coding and digital literacy programs early on and fostering partnerships with international institutions to enhance curriculum and faculty development. Additionally, creating competitive research institutions and incentivizing innovation can drive scientific advancement and technological breakthroughs.

### Fiscal incentives for innovation

To stimulate private-sector investment in innovation, governments should introduce targeted fiscal incentives for companies that invest in research and development (R&D). Measures could include tax credits for businesses dedicating a specific percentage of revenue to R&D and offering accelerated depreciation for investments in innovative technologies and equipment. Additionally, governments should create regulatory environments that are conducive to innovation, such as streamlined approval processes for new products and services.

### Private sector involvement

Increasing private sector involvement can be achieved through tailored public-private partnership (PPP) frameworks for STI projects. These partnerships can promote collaboration between academia, industry, and government to collectively address national challenges. Innovation hubs that bring together researchers, entrepreneurs, and established companies can further foster an ecosystem of knowledge transfer and commercialization. Additionally, mentorship programs linking startups with experienced companies will enhance innovation by providing the necessary guidance and resources for success.

### Strengthening regional collaboration

Establishing a Central American STI Network would enable resource pooling, knowledge sharing, and collaboration across the region. A digital platform could connect researchers, facilitate project collaboration, and streamline knowledge dissemination. A regional mobility program for researchers and students would further support knowledge exchange, while annual STI conferences would showcase regional research and foster partnerships. Joint funding mechanisms for cross-border research on shared challenges, along with regional centers of excellence in fields like tropical diseases and sustainable agriculture, would enhance collective STI capabilities. Integrating with ACAL-Conecta could also join Central American researchers with broader Latin American networks, creating economies of scale and expanding research impact. To build on these initiatives, the model of Nicaragua's Biotechnology Conferences, organized by the UCA Molecular Biology Center, offers an effective example (Huete-Pérez et al., 2012). Since 2000, these biennial events have connected academia, industry, and government, promoting biotechnology research, addressing regional needs, and fostering innovation opportunities. This approach strengthens national and regional scientific networks, advancing public-private partnerships and research infrastructure. Adopting a similar model within the proposed Central American STI Network could significantly enhance collaboration and development across the region.

By implementing these specific actions, governments can create an environment conducive to STI development, fostering a culture of innovation and driving economic growth. Regular monitoring and evaluation of these initiatives will be essential to ensure their effectiveness and facilitate adjustments as needed.

While these broad strategies provide a regional framework, it's crucial to recognize that each country faces unique challenges requiring tailored approaches. Guatemala needs to prioritize environmental sustainability initiatives, while Honduras should focus on implementing consistent STI policies. El Salvador's primary focus should be on improving educational quality, and Nicaragua must work toward restoring academic freedom. Costa Rica can build on its strengths by further leveraging its thriving startup ecosystem, while Panama should develop strategies to retain its PhD graduates and curb brain drain.

By implementing these interconnected strategies—educational reform, collaboration, government support, and country-specific initiatives—Central America can pave the way for a brighter future, harnessing the power of STI to drive sustainable development and improve the lives of its citizens.

## From challenges to opportunities

Despite challenges, collaborative social science research across Nicaragua, El Salvador, and Guatemala highlights the transformative potential of regional cooperation. Costa Rica's successful startup scene in sectors like renewable energy and biotechnology further demonstrates the region's potential for innovation.

To unlock the potential of STI in Central America, strategic policy frameworks tailored to each country's needs are crucial. Sustained financial investment in research and development is essential for long-term growth. Currently, Central American countries invest only a small percentage of their GDP in R&D compared to developed nations. Increasing this investment to around 1% of GDP could be a realistic target to foster regional innovation, attract talent, and create high-value jobs.

While developed economies like the United States, the European Union, and South Korea allocate 2–4% of GDP to R&D, Latin American and Caribbean countries fall behind, with regional investment decreasing from 0.7% to 0.6% of GDP between 2015 and 2021 (ECLAC, 2024). According to UNESCO, Costa Rica invested 0.39% of its GDP in R&D in 2018, El Salvador 0.16%, Panama 0.13%, Guatemala 0.03%, and Honduras 0.04% (Lewis et al., 2021), while recent data for Nicaragua remains unavailable. This stark disparity highlights the urgent need for increased R&D funding to drive sustainable development and innovation in Central America.

Boosting public R&D investment not only supports technological advancement but also strengthens economic growth, job creation, and quality of life. By aiming for 1% of GDP in R&D spending, Central American countries can enhance their global competitiveness and promote inclusive economic growth that benefits a broader spectrum of society.

Furthermore, fostering collaboration between universities, research institutions, and the private sector is vital to leverage resources and expertise. By addressing regional challenges, implementing strategic policies, and fostering collaboration, Central America can harness science and technology to drive sustainable development and improve the lives of its citizens.

## Discussion

The dynamic landscape of Science, Technology, and Innovation (STI) policies in Latin America underscores an urgent demand for robust analytical frameworks to address national challenges. There is a discernible shift from traditional National Innovation System (NIS) approaches to problem-oriented paradigms, advocating for holistic policies that intricately consider the systemic nature of challenges (Ghazinoory et al., 2020). This shift stresses the adaptability of effective STI policies, placing particular emphasis on governance mechanisms to navigate complexity and highlighting the indispensable role of end-users in the policymaking process (Alvarez et al., 2020).

Central America's National Innovation Systems are evolving, presenting both opportunities and challenges for sustainable development. Looking ahead, the region's STI development hinges on several critical factors: strengthening institutional frameworks, fostering sustainable funding mechanisms, and most importantly, engaging youth in STI leadership. This focus on youth engagement is particularly crucial as it represents both an emerging trend and a key strategy for long-term development. These efforts must carefully balance innovation goals with social cohesion needs, recognizing that sustainable development requires both technical advancement and social stability. The diverse experiences across Central America, from the more advanced systems of Costa Rica and Panama to the emerging frameworks in other nations, offer valuable insights for developing nations facing similar challenges in building effective National Innovation Systems.

The responsibility of science communicators is underscored, emphasizing the need for scientists to engage in culturally relevant communication and research translation (Cumba García, 2020, 2021). Importantly, complex, and adaptive actions are needed to address intricate problems, with a particular emphasis on the teachability and learnability of science diplomacy and the science-policy interface. Collectively, this discussion illuminates the multifaceted nature of STI policies in Latin America, advocating for adaptive, inclusive, and holistic approaches to drive transformative advancements and, crucially, to empower the next generation of scientists.

Drawing insights from the Mexican experience, the discussion extends to the pivotal role of educational institutions in nurturing emerging scientists. Amendments to Science, Technology, and Innovation Policies (STIP) in Mexico are underscored, emphasizing the significance of the Science Policy Interface as a social process involving scientists and policymakers (Van den Hove, 2007). The discussions identified a transformative shift from “science-based” to “science-informed” policies, challenging cultural perceptions and emphasizing inclusivity in scientific input in policy considerations.

Concrete examples include the amendment for incentives to create spin-offs based on science (Hernandez-Mondragon et al., 2016) and the Mexico City Science Policy Fellowship. The fellowship provides researchers with a valuable opportunity to directly influence policymaking at both national and international levels (Hernández-Mondragón, 2022). The discussions placed significant importance on the involvement of scientists in evidence-informed policymaking, stressing proactive outreach and engagement in third-sector organizations. Strategies for science policy career preparation, including fellowships, partnerships, and policy and diplomacy training, are relevant (John et al., 2023).

The Mexican experience highlights the importance of adapting STI policies to the evolving landscape (Natera et al., 2019). Central American countries could consider adopting a flexible policy framework that can accommodate changes in technology and address emerging challenges (Hernández Mondragón and Castañeda Hernández, 2023). This adaptability should extend to governance mechanisms, ensuring the policies remain effective in navigating complexity. Encouraging active collaboration and dialogue between the scientific community and policymakers can lead to more informed and inclusive policies (Dutrénit et al., 2018).

Examining the challenges and lessons in Colombian science and technology offers insights applicable to Central America. Historically reliant on imported technology, Colombia's pivotal step in 1968 with the creation of Colciencias marked a commitment to impactful policies. Successful initiatives, such as the Caldas Network and technology development centers, highlight positive strides (Montoya and Rivera, 2013; Chaparro et al., 2016). However, the need for skilled individuals remains a challenge, emphasizing the importance of investing in human capital.

Recent shifts, including Colciencias becoming a ministry in 2019 and efforts to promote South-South collaboration, are notable. Yet, projected budget reductions for science and technology in 2024 and issues like companies prioritizing technology over knowledge pose obstacles. Comparisons with developed countries underscore the urgency of increased R&D investment. Advocating for long-term programs, human talent development, consistent government support for research institutions, and effective governance are crucial for overcoming future challenges.

Despite the Colombian Academy of Sciences' instrumental role, the country's STI investment remains modest at 0.29% of GDP (World Bank, 2024), contrasting sharply with the US. This serves as a reflective guide for Central America, emphasizing the need for strategic and sustained investments in science and technology.

The experiences of other Latin American countries offer valuable insights into effective STI policy implementation. Argentina's strategic focus on key sectors such as biotechnology and nuclear energy, coupled with its emphasis on international collaborations, has significantly enhanced its scientific output and technological competitiveness (Albornoz and Gordon, 2011). Chile has emerged as a regional leader in astronomy by investing in world-class facilities, which has not only advanced scientific knowledge but also boosted science education and tourism (Guridi and Pertuze, 2020). Brazil's approach of fostering public-private partnerships in research and development has led to notable innovations, particularly in sustainable agriculture and energy production (De Negri and Squeff, 2016). These diverse strategies demonstrate the importance of targeted investments, international cooperation, and industry collaboration in driving STI progress. By examining these varied approaches alongside the experiences of Mexico and Colombia, we can gain a more comprehensive understanding of effective STI strategies in the Latin American context, providing valuable lessons for other countries in the region seeking to enhance their innovation ecosystems.

Furthermore, Central America requires with urgency comprehensive interventions to mitigate the escalating food insecurity crisis in Central America, where food security has reached its highest rate in the past two decades, affecting 19 million people (10.6% of the population). The Caribbean and South America also experience significant food insecurity, with 7 million and 33.7 million people affected, respectively (World Bank, 2021). Recent studies, such as the one by Benites-Zapata et al. (2021), highlight the severe impact of the COVID-19 pandemic on food security in Latin America and the Caribbean, emphasizing the role of sociodemographic factors and pandemic-related variables.

Addressing food security challenges in Central America requires a strategic focus on technological innovation within agri-food systems, involving both public and private sector efforts. Currently, public investment in agriculture is insufficient, and although private sector investment is higher, it still falls short of what is needed. Agricultural technology, predominantly designed for high-income countries, has proven less effective in regions like Central America, where only 58% of global agricultural productivity is achieved. Therefore, it is essential to redirect existing agricultural investments to address local challenges, while simultaneously increasing overall investment levels to meet the growing global demand for food and the rising production costs.

Science, Technology, and Innovation (STI) policies play a central role in this transformation, with governments needing to lead through cost analyses to make informed decisions on sectors that require increased investment or strategic reallocation. Funders also emerge as key stakeholders, capable of facilitating and supporting these shifts. The discussion highlights the urgent need for a nuanced and strategic approach to tackling food security challenges, harnessing technological innovations supported by robust STI policies to offer sustainable and inclusive solutions that can address regional challenges while contributing to global agricultural productivity.

The workshop also explored methods for evaluating key STI policies in Central America, specifically focusing on their applications in energy conservation. These encompass energy-efficient appliances, demand-side management, and sector-specific energy plans.

Highlighting the significance of innovative evaluation methodologies, the workshop addressed STI policies targeting energy conservation and behavior change in the Central American context. The central role of policy interventions in energy-related sectors like buildings and transportation was stressed to manage increasing energy consumption and achieve regional energy transition goals. The workshop delved into practices such as using Randomized Control Trials to establish causal links between innovation strategies and social impacts, revealing challenges related to voluntary participation in STI policies. Real-time appliance-level, environment, and health-based information strategies were found to be more effective than monetary savings information in driving energy conservation (Asensio and Delmas, 2015).

Throughout the discussions, there was consistent emphasis on the importance of policy interventions in critical energy-related sectors. The workshop advocated for leveraging behavioral strategies through information technologies as effective components of sustainable development pathways. Crucially, these strategies do not demand extended lead times typical of new capital investments in energy infrastructure. These insights offer valuable considerations for shaping impactful policies in the context of Central America's energy conservation efforts, advancing our understanding of the effectiveness of information-based conservation policies.

The workshop delved into the vital concepts of capacity and resilience in community development, emphasizing the imperative to train decision-makers and practitioners. This is essential to address the dynamic nature of communities, requiring innovative capacity-building strategies and technologies.

Human development's linkage to global peace and security necessitates successful and resilient innovation ecosystems. These ecosystems should benefit people by ending poverty and hunger, ensuring dignity and equality. They should also protect the planet, preserving natural resources and climate for future generations. Achieving these goals requires solid global partnerships with businesses, contributing to prosperous lives in harmony with nature for more enduring peace.

Central America's aim is to promote sustainable, healthy, stable, equitable, safe, and prosperous communities. However, this is challenging in our fast-paced, uncertain, ambiguous, and complex world with interconnected processes and variables.

The workshop underscored Central America's potential to be a regional leader in Engineering, Science, Technology, and Innovation (ESTI) education. To achieve this, developing strength and capacity in ESTI is crucial for addressing sustainable development needs and fostering a global knowledge and skill-based economy. The focus is on training today's youth with the necessary skills to tackle global challenges. Initiatives such as Engineers without Borders in San Pablo, Belize, exemplify collaborative efforts between university students, engineers, and business owners to address challenges. The goal is to train a new generation of engineers and scientists not just as technical providers but as change-makers, peace-makers, and facilitators of sustainable human development. This training is vital for handling the spectrum from crisis to development, especially in the face of increased disasters over the past 20 years.

To formulate regional plans for ESTI in Central America, understanding how development problems are addressed by various constituencies (academia, industry, government, and civil society) is crucial. Training workshops can facilitate the review of these problems and the development of educational curricula addressing both regional and in-country challenges.

The discussion prompted reflection on integrating science into development problem-solving, echoing Einstein's perspective that the significant problems of today require a higher level of thinking than when they were created.

### Workshop regional and global significance

Central America, particularly in the Northern Triangle, faces persistent challenges leading to migrations driven by issues like poverty, violence, and corruption. The perilous journey through the Darién jungle poses threats, impacting migrants' wellbeing with risks ranging from injuries to violence. Territorial disputes, as seen with Nicaragua, add complexity to the region's dynamics. These formidable difficulties have profound implications for sustainable development in the area, hindering progress and resilience.

Science diplomacy may serve as a potent means to connect nations amid political tensions, fostering collaboration and mutual understanding, even in the face of strained relations and a tumultuous history (García et al., 2024). Utilizing science diplomacy emerges as a strategic approach to address these multifaceted problems, promoting collaborative solutions for poverty alleviation, violence prevention, and conflict resolution. International scientific cooperation, including exchanges, joint research initiatives, and knowledge-sharing, becomes instrumental in evidence-informed policymaking and sustainable development practices. This approach enables the region to harness collective expertise, fostering resilient innovation systems that tackle the root causes of migration and contribute to long-term stability and prosperity.

The workshop facilitated collaboration among scholars, policy experts, and researchers from Central America and the United States, exemplifying the application of science diplomacy to address common global and regional challenges. These challenges include sustainable development and advancements in science, technology, and innovation (STI). The engagement of experts from diverse backgrounds promoted international cooperation and understanding, showcasing the role of science in diplomacy by providing advice and evidence for decision-making in regional affairs. This collaborative model serves as an example of how countries with varying levels of scientific development can unite to enhance global knowledge sharing. For developed nations, it offers insights into supporting less-developed nations in their scientific endeavors, while developing nations can benefit from the experiences of more advanced counterparts, contributing to a balanced global science landscape.

The findings from this workshop can inform international organizations and development agencies working with less developed countries worldwide. Lessons about crafting effective science, technology, and innovation (STI) policies in resource-constrained environments can be applied globally, encouraging more countries to prioritize STI as a catalyst for progress.

## Conclusion

The international workshop served as an effective forum for fostering discussions, presentations, and the exchange of knowledge pertaining to science, technology, and innovation (STI) policies, innovation systems, and scientific advancements in Latin America, focusing on Central America. Its overarching goal was to facilitate and expedite sustainable growth while enhancing the quality of life in the region. The valuable insights and recommendations that emerged from the workshop carry the promise of fortifying STI policies and innovation systems in Central America, thus advancing the cause of sustainable development. The implications of this workshop extend beyond regional borders. They bear relevance to the broader context of global science diplomacy, and concurrently address the specific challenges confronted by Central America.

Central America grapples with limited resources and inefficient national innovation systems. This workshop brings together regional stakeholders to address these challenges. Lessons from this event can help Central American nations better utilize their available resources for STI. The emphasis on diversifying the research landscape is particularly relevant to Central America, where many nations have faced stagnation in researcher numbers. By following the example of countries like Costa Rica and Panama, which prioritize research diversity, Central American nations can revitalize their research ecosystems. The workshop highlighted a critical issue in Latin America, also pertinent to Central America: the gap between the scientific community and policymakers. To address this, the establishment of governmental scientific advisory bodies was proposed, aiming to enhance the relevance and impact of STI policies in the region. See Table 3.

The discussions emphasized the transformative potential of the youth, the necessity to comprehend scientists' perspectives, and the influential role of leadership and diaspora in utilizing knowledge for national benefit. Challenges, including a reliance on individuals over institutions, the weakening of universities, and the departure of scientists, were acknowledged. Additionally, the dialogue underscored the importance of resilience, motivation, and presenting a compelling vision for the future. These aspects transcend political shifts, fostering interdisciplinary, long-term pursuits that inspire hope and progress.

Key takeaways from the workshop emphasize the imperative for Central American nations to invest in science-related education to cultivate the essential human capital for tackling climate-related challenges. The discussion advocates for proactive measures such as improving educational quality, incorporating scientific values into cultural narratives, and fostering a distinct national identity to attract knowledge and talent.

The workshop emphasized the significant role of biotechnology, especially in healthcare, as a potentially crucial sector for the region. Leveraging the strengths of biotechnology could further advance the bioeconomy, encompassing bioenergy, waste reuse, and agro-industry. This strategic shift toward the bioeconomy offers a pathway to tackle challenges associated with economic dependence on primary commodities (agriculture, mining, fossil resources), fostering diversification and structural change while mitigating instability stemming from price volatility.

Furthermore, the workshop emphasized the significance of adaptation to climate change and improved energy conservation efficiency. Given that Central America is highly vulnerable to climate change, leveraging science, technology, and innovation is vital for achieving sustainable development. The workshop's focus on energy conservation and behavior change has direct implications for Central America's energy transition. As per capita energy consumption increases, Central American countries need to adopt energy-efficient technologies and develop clear STI strategies for a sustainable future.

The workshop served as a catalyst for global science diplomacy, bridging the gap between developed and developing nations. Its findings and recommendations can significantly impact Central America by addressing resource constraints, promoting diversified research, strengthening science-policy connections, and providing guidance for sustainable development in the face of climate change. The workshop demonstrated how collaborative global efforts can drive meaningful progress in science, technology, and innovation.

In organizing this workshop, we aimed to ensure broad representation of perspectives, positions, and visions from various stakeholders across the region, including scholars, academies, and representatives from diverse countries. However, we acknowledge that the scope of our discussions is inherently limited by the financial resources available to convene stakeholders from all the nations involved. Despite these constraints, we believe this work offers valuable contributions to the existing literature on STI policies and development in Central America. It is our hope that the insights and recommendations presented here will not only inform future policy decisions but also inspire further research and collaboration in this critical area.
